# The contribution of respiratory pathogens to fatal and non-fatal respiratory hospitalizations: a pilot study of Taqman Array Cards (TAC) in Kenya

**DOI:** 10.1186/s12879-017-2694-0

**Published:** 2017-08-25

**Authors:** Henry N. Njuguna, Sandra S. Chaves, Gideon O. Emukule, Bryan Nyawanda, Victor Omballa, Bonventure Juma, Clayton O. Onyango, Joshua A. Mott, Barry Fields

**Affiliations:** 1 Centers for Disease Control and Prevention, Nairobi, Kenya; 2 Kenya Medical Research Institute, Nairobi, Kenya

**Keywords:** Respiratory pathogens, Respiratory illness, Multi-pathogen Taqman array cards

## Abstract

**Background:**

Respiratory diseases cause substantial morbidity and mortality worldwide, with sub-Saharan Africa bearing the greatest burden. Identifying etiologies of respiratory disease is important to inform cost effective treatment, prevention and control strategies. Testing for all of the different pathogens that are potentially associated with respiratory illnesses is challenging. We piloted the use of a multi-pathogen respiratory Taqman Array Cards (TAC) to identify pathogens in respiratory samples collected from non-fatal and fatal cases and their matched asymptomatic controls.

**Methods:**

This is a case control study comparing viral and bacterial pathogens detected among non-fatal and fatal cases to those detected among age and time matched asymptomatic controls. We used McNemar’s test to compare proportions of pathogens detected among cases (non-fatal and fatal) to their matched asymptomatic controls. We used Mann-Whitney test to compare the distribution of median Cycle threshold (Ct) values among non-fatal and fatal cases to their corresponding asymptomatic controls.

**Results:**

There were 72 fatal and 72 non-fatal cases matched to 72 controls. We identified at least one pathogen in 109/144 (76%) cases and 59/72 (82%) controls. For most pathogens, the median Ct values were lower among cases (fatal and non-fatal) compared to asymptomatic controls.

**Conclusions:**

Similar rates of pathogen detection among cases and controls make interpretation of results challenging. Ct-values might be helpful in interpreting clinical relevance of detected pathogens using multi-pathogen diagnostic tools.

**Electronic supplementary material:**

The online version of this article (10.1186/s12879-017-2694-0) contains supplementary material, which is available to authorized users.

## Background

Respiratory illnesses associated with bacteria, fungi or viral infections cause substantial morbidity worldwide. Specific etiology cannot be distinguished on basis of clinical presentation alone. Understanding the relative contribution of different pathogens to severe respiratory illnesses could be an important tool to inform and prioritize specific clinical interventions or public health policies. Nonetheless, testing for all of the different pathogens that are potentially associated with respiratory illnesses is challenging.

Molecular-based multi-pathogen diagnostic tests such as Taqman Array cards (TAC) may offer some resolution to the diagnostic challenges of respiratory illnesses, especially in the setting of outbreaks of unknown etiology. These tests are capable of detecting a broad range of pathogens by multiplexing polymerase chain reaction (PCR), and have a short turn-around-time of approximately 3 h [1]. We piloted the use of respiratory TAC to identify pathogens in nasopharyngeal (NP) and oropharyngeal (OP) specimens collected from hospitalized patients with respiratory illness (fatal and non-fatal cases) and corresponding matched asymptomatic controls. The hypothesis was that as we moved through the spectrum of disease severity (from asymptomatic to fatal cases), we would see changes in the types and frequency of pathogens detected that could assist with interpretation of disease etiology.

## Methods

### Participants and definitions

Clinical data on patients’ specimens collected from 1st Aug 2009 through 31st Jul 2011 at Siaya County Referral Hospital (Siaya CRH) and Lwak Mission Hospital (Lwak MH) were obtained. Both hospitals are located in rural western Kenya, and surveillance activities are operated by the Kenya Medical Research Institute (KEMRI) in collaboration with the U.S. Centers for Disease Control and Prevention in Kenya (CDC-Kenya) [2, 3]. At Siaya CRH, for consenting patients hospitalized with acute respiratory illness (cases) demographic data were collected using structured questionnaires if at least one of the following symptoms had been present during the previous 14 days: cough, difficulty breathing, sore throat, or chest pain. In addition, NP/OP specimens were collected for TAC testing. Cases were further classified as non-fatal if they were discharged from hospital alive or fatal if they died during hospitalization. We also enrolled asymptomatic controls among those visiting the outpatient clinic at Lwak MH due to non-infectious conditions. Reasons for outpatient visit could include drug refills, vaccination services or well-visits; persons accompanying sick relatives were also approached for enrollment as control. Controls were excluded if they reported respiratory symptoms, fever or diarrhea within 14 days prior to their clinic visit [4]. Lwak MH is located at approximately 30 km from Siaya CRH, serving a similar population [5]. Initially, we identified non-fatal cases and matched them individually with fatal cases based on age and date of admission (within +/− 30 days) at a ratio of one-to-one. Then we identified a similar number of asymptomatic controls within the same time-frame as the cases and matched them in similar fashion (based on age and date of admission) to the previously matched non-fatal and fatal cases. The age-matching was done considering up to 4 months difference for those aged <18 years, and a 5 year-band difference for those ≥18 years.

### Laboratory methods

NP/OP swabs were combined in a single cryovial containing 1 ml viral transport media (VTM). These specimens were transported on the same day in cool boxes at 2-8 °C to the KEMRI laboratory (Kisumu, Kenya) where they were stored at −80 °C until processed. Fifty microliters of NP/OP specimen was tested using respiratory TAC for viral targets [influenza virus type A, influenza virus type B, respiratory syncytial virus (RSV), parainfluenza virus type I (PIV-1), PIV-2, PIV-3, Human metapneumovirus (HMPV), Rhinovirus /Enterovirus (RV/EV), and Adenovirus (AV)] and bacterial targets [*Streptococcus pneumoniae, Haemophilus influenzae* pan*, Mycoplasma pneumoniae* and *Bordetella pertussis*] [6] [for more details, see Additional file 1: Fig. S1 (Schematic diagram of the two versions of Taqman array cards (TAC) used in the study) in supplementary material]. A positive result in TAC for each pathogen was defined as cycle threshold (Ct) values <40.

### Statistical analysis

We stratified the study population by age group and calculated the prevalence of each virus detected from NP/OP specimens collected from non-fatal and fatal cases and asymptomatic controls. We used McNemar’s test to compare the proportions of pathogens detected among cases (non-fatal and fatal) to their matched asymptomatic controls. We used odds ratios (OR) to determine pathogens associated with cases (non-fatal and fatal) compared to controls. We used the median Ct value as a proxy of pathogen density in the NP/OP specimen [7], using the Mann-Whitney test to compare the distribution of the median Ct values between non-fatal and fatal cases and their corresponding asymptomatic controls separately for each pathogen identified. All statistical analysis was carried out using Stata version 13.0 (StataCorp. 2013. Stata Statistical Software: Release 13. College Station, TX: StataCorp LP) and *p*-values <0.05 were considered to statistically significant.

## Results

There were equal number of fatal and non-fatal cases and matched controls (*n* = 72 each). Of the total study-patients (*n* = 216), 102 (47%) were aged <5 years and 114 (53%) were ≥5 years. For each age category, the median age was 1.5 years (range 3 - 59 months) for those <5 years and 36 years (range 5 - 90 years) for those ≥5 years. Among non-fatal cases, 61 (85%) presented with cough, 40 (56%) with difficulty in breathing, 17 (24%) with chest pain and 10 (29%) with sore throat (these symptoms were not mutually exclusive). Among fatal cases, 63 (88%) presented with cough, 45 (63%) with difficulty in breathing, 19 (26%) with chest pain, and 13 (41%) with sore throat.

Using TAC, we detected at least one pathogen from 109/144 (77%) cases. There was no difference in pathogen distribution among non-fatal and fatal cases based on the presenting symptoms. Overall, among all cases, there was a higher percent of viral co-detection (11% had 2 viruses and 6% had ≥3 viruses detected) compared to bacteria co-detection (7% had 2 bacteria detected and 1% had ≥3 bacteria detected). There were more viral co-detections among non-fatal cases, with 6(8%) having ≥3 viruses detected compared to 3(4%) among fatal cases; but total number of detected pathogens were similar in the two groups (*n* = 54 vs *n* = 55 among non-fatal and fatal cases respectively). Children <5 years had a higher percent of pathogens detected than those ≥5 years: 100% vs 52% among non-fatal, and 88% vs 66% among fatal cases, respectively (Additional file 1: Table S1). We detected at least one pathogen from 59/72 (82%) asymptomatic controls with higher co-detection of bacteria (44% had 2 bacteria) compared to viruses (15% had 2 viruses). Only 4/72 (6%) asymptomatic controls developed an illness after sample collection. One developed respiratory symptoms 6 days after being swabbed (originally had rhinovirus detected in the study sample); one developed fever 3 days after being swabbed (had no pathogens detected); one developed unspecified non-respiratory symptoms 3 days after sample collection (had enterovirus, *Haemophilus influenzae* and *S. pneumoniae* detected in the study sample); and one developed respiratory symptoms 1 day after the NP/OP sample being collection (had no pathogens detected).

**Fig. 1:**
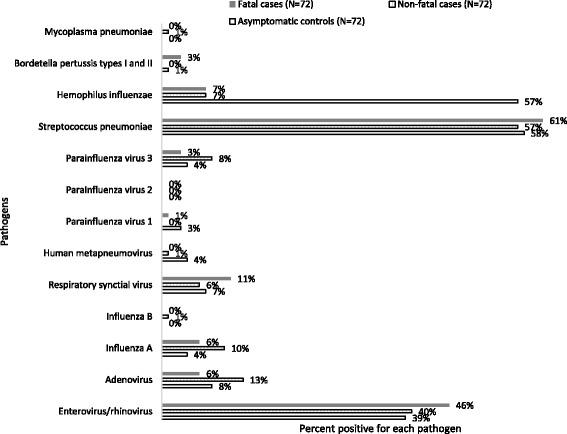
Distribution of viruses and bacteria detected using Taqman array cards (TAC) among 72 non-fatal cases, 72 fatal cases, and 72 matched asymptomatic control

**Fig. 2:**
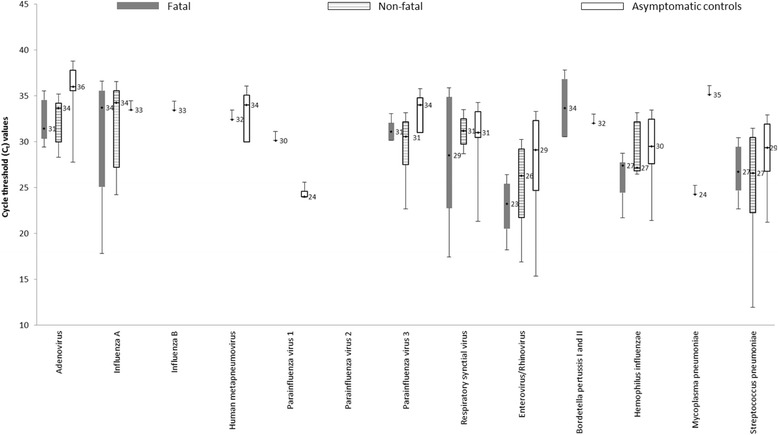
Box plot showing cycle threshold (Ct) values for agents targeted by Taqman Array Card (TAC) among 72 non-fatal cases, 72 fatal cases and 72 asymptomatic controls. Boxes show the median (dot and number) and the 25th and 75th percentiles and bars show the 10th and 90th percentiles. *Pathogens whose median Ct value is significantly lower among non-fatal or fatal cases compared to asymptomatic controls using Mann-Whitney test (Enterovirus/Rhinovirus median Ct: fatal-cases (23.23) vs asymptomatic controls (29.10), *p* value < 0.01; *S.pneumoniae* median Ct: non-fatal cases (26.60) vs asymptomatic controls (29.34), *p* value <0.01)

Enterovirus/rhinoviruses were the most commonly detected viruses: 40% among non-fatal cases, 46% among fatal cases and 39% among asymptomatic controls (Additional file 1: Table S1). There was no difference in enterovirus/rhinovirus detection between non-fatal and asymptomatic controls (*p* = 0.87) and fatal and asymptomatic controls (*p* = 0.40). The most commonly detected bacteria was *S. pneumoniae*: 57% among non-fatal cases, 61% among fatal cases and 58% among asymptomatic controls. There was no difference in *S.pneumoniae* detection between non-fatal and asymptomatic controls (*p* = 0.09) and fatal and asymptomatic controls (*p* = 0.08) (Fig. 1). *H. influenzae* were less likely to be detected among non-fatal cases (OR 0.08, 95% CI 0.02-0.25) and fatal cases (OR 0.05, 95% CI 0.12-0.22) compared to their respective asymptomatic controls (Additional file 1: Table S2). However, for almost all more frequently detected pathogens (i.e., adenovirus, PIV-3, RSV, enterovirus/rhinovirus, *Streptococcus pneumoniae,* and *Haemophilus influenzae*), median Ct values were lower for cases (fatal and non-fatal) than controls, though these differences were not statistically significant (Fig. 2). Enterovirus/rhinovirus had significantly lower median Ct values among fatal cases compared to asymptomatic controls (*p* < 0.01); and *S. pneumoniae,* had significantly lower Ct values among non-fatal cases compare to asymptomatic controls (*p* < 0.01).

## Discussion

We detected multiple pathogens in NP/OP specimens collected among non-fatal and fatal cases and asymptomatic controls. Careful interpretation of results is needed since over 80% of participants enrolled as asymptomatic controls had at least one pathogen identified. Ct values may play an important role when assessing etiological role of pathogens detected in similar rates among cases and controls.

Enterovirus/rhinovirus, RSV and adenovirus were the leading viral pathogens detected from non-fatal and fatal cases, similar to previous reports [4, 8, 9]. *S. pneumoniae* was the leading bacterial pathogen detected in both non-fatal and fatal cases (60%). These rates were within the range observed from other studies, i.e., 55-75% [8, 10]. We did not find substantial differences in pathogen detection in cases (fatal and non-fatal) compared to controls. Other studies have shown pathogens such as influenza virus, RSV, rhinovirus, HMPV and *Streptococcus pneumoniae* being detected more frequently in cases compared to asymptomatic controls [9, 11, 12]. In fact, in our study, *H. influenzae* had a 5 fold increase in detection rates among asymptomatic controls compared to cases – even considering bacteria carriage as common in the population, this may need further investigation. The 14 day symptom-free period required for controls could have been too short, some patients may be convalescent, coming for drug refills. On the other hand, cases could have the distribution of bacterial pathogens in the respiratory tract altered by previous antibiotic treatment [13].

Despite the lack of statistical significance, the median Ct values for adenovirus, PIV-3, RSV, enterovirus/rhinovirus, *Streptococcus pneumonia,* and *Haemophilus influenzae* were lower among cases (fatal and non-fatal) compared to asymptomatic controls; this could suggest an etiologic contribution to the clinical presentation. Previous studies have demonstrated that Ct values could be a proxy for assessing concentration of pathogens and lower values have been associated with disease severity [7]. Both fatal and non-fatal cases positive for enterovirus/rhinovirus and *S. pneumoniae* had significantly lower median Ct values than asymptomatic controls. Assuming that Ct values could be a proxy for pathogen density, enterovirus/rhinovirus and *S. pneumoniae* could have played a role in the pathogenesis of respiratory illness among case-patients. High density of *S. pneumoniae* in the nasopharynx, for instance, has been associated with pneumonia [14]. For future studies using multi-pathogen diagnostic platform, the strength of association between pathogen detected with respiratory illness may have to be assessed relative to pathogen loads in order to infer etiology.

There were a number of limitations to this study. NP/OP swabs are non-sterile-site specimens. Using a highly sensitive molecular technique on non-sterile specimens can detect small amounts of nucleic material even in asymptomatic individuals who are either incubating or recovering from infection [15]. Our case definition was quite broad. It is possible that some patients presented with other illnesses such as malaria (endemic in this area) or with nonspecific respiratory symptoms were misclassified as having a respiratory disease. This may have reduced the chances of detecting respiratory disease pathogens among the cases. The low sample size in our study does not afford the statistical power needed to determine an association between most pathogens detected and respiratory disease, despite the trend toward some pathogens of lower Ct values (i.e., high concentration) amongst non-fatal and fatal cases compared to controls.

## Conclusions

Multi-pathogen molecular diagnostics such as TAC can be useful for investigation of respiratory outbreaks of unknown origin and to assess the distribution of pathogens in a defined population. However, the attribution of pathogens as a cause of respiratory disease using this technology needs to be further evaluated. This study supports previous published studies suggesting the use of Ct values to define clinically relevant pathogen concentrations. Further studies are warranted to investigate and establish appropriate pathogen-specific Ct cutoff values indicative of disease.

## Additional file

Additional file 1: Figure S1. Schematic diagram of the two versions of Taqman array cards (TAC) used in the study. **Table S1.** Viral and bacterial pathogens detected using Taqman array cards (TAC) among non-fatal and fatal cases and asymptomatic controls, by age group, western Kenya, 2009-11. **Table S2.** Distribution of respiratory pathogens among cases (non-fatal and fatal) and corresponding asymptomatic controls in rural western Kenya, 2009-11. (DOCX 614 kb)

## Abbreviations

AV: Adenovirus
CDC: Centers for Disease Control and Prevention
CRH: County Referral Hospital
Ct: Cycle threshold
EV: Enterovirus
HMPV: Human metapneumovirus
KEMRI: Kenya Medical Research Institute
MH: Mission Hospital
NP: Nasopharyngeal
OP: Oropharyngeal
OR: Odds ratio
PCR: Polymerase chain reaction
PIV: Parainfluenza virus
RSV: Respiratory syncytial virus
RV: Rhinovirus
TAC: Taqman Array Cards
VTM: Viral transport media
