# Supplementary material for: The contribution of respiratory pathogens to fatal and non-fatal respiratory hospitalizations: a pilot study of Taqman Array Cards (TAC) in Kenya
Source: BMC Infect Dis. 2017 Aug 25;17:591. doi: 10.1186/s12879-017-2694-0 (PMC5574104; doi:10.1186/s12879-017-2694-0)
Supplement: Additional file 1: Figure S1. — Schematic diagram of the two versions of Taqman array cards (TAC) used in the study. Table S1. Viral and bacterial pathogens detected using Taqman array cards (TAC) among non-fatal and fatal cases and asymptomatic controls, by age group, western Kenya, 2009-11. Table S2. Distribution of respiratory pathogens among cases (non-fatal and fatal) and corresponding asymptomatic controls in rural western Kenya, 2009-11. (DOCX 614 kb) [file 12879_2017_2694_MOESM1_ESM.docx]

**Supplementary material**

**
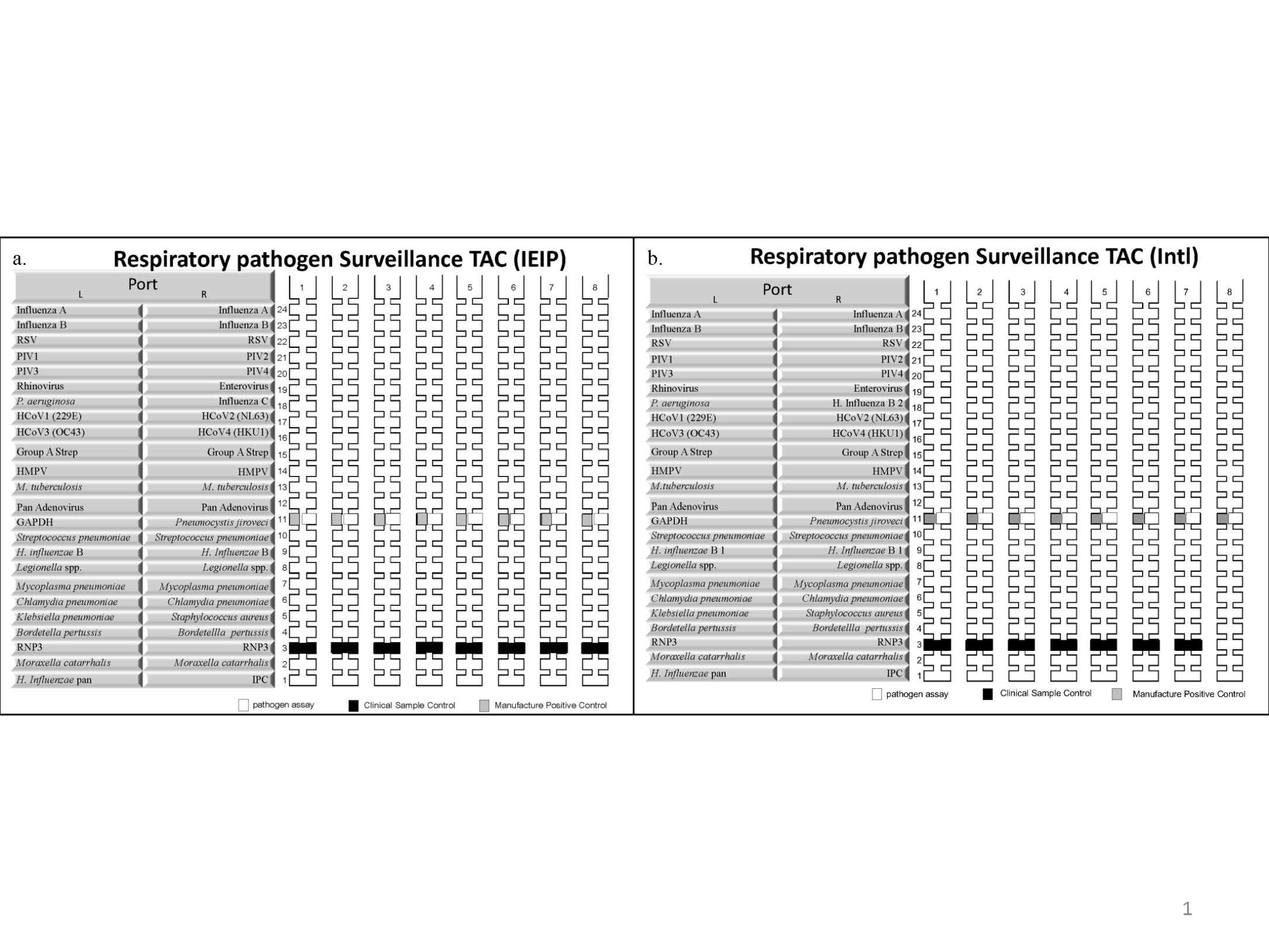
**

Supplementary Figure S1. Schematic diagram of the two versions of Taqman array cards (TAC) used in the study

Note: GAPDH- Glyceraldehyde-3-Phosphate Dehydrogenase; RNP-human RNase P gene.

**Supplementary figure legend**

Due to the limited number of respiratory TAC available, we used two different versions of the respiratory TAC cards; version (a) and (b) (Figure S1). Initially, we used version (a) to test specimens collected from non-fatal and fatal cases. Later on we identified asymptomatic controls and matched them to non-fatal and fatal cases and used version (b) to test specimens collected from them. The difference between these two versions was that influenza type C in the version (a) card was replaced by *Hemophilus influenzae* type B_2 in the version (b) card. In this study, we considered only pathogens that were contained in both cards. These targets included: 10 viral targets [influenza virus type A, influenza virus type B, respiratory syncytial virus (RSV), parainfluenza virus type I (PIV-1), PIV-2, PIV-3, Human metapneumovirus (HMPV), Rhinovirus (RV), Enterovirus (EV), Human Coronavirus (HCov), and Adenovirus (AV)] and 4 bacterial targets [*Streptococcus pneumoniae, Hemophilus influenzae* pan*, Mycoplasma pneumoniae and Bordetella pertussis*. TAC cards are based on a 384 well card with PCR primers and probes lyophilized in each well for 384 singleplex PCR reactions. Each PCR reaction consisted of 50uL 2X AgPath-ID One-step RT-PCR, 4uL 25X enzyme mix and 46uL of individual TNA prior to running on the Applied Biosystems ViiA7 Real-Time PCR system (Life Technologies, Foster City, CA, USA). Thermal cycling conditions included 45°C for 10 min for reverse transcription, 94°C for 10 min for polymerase activation and 45 cycles of 94°C for 30s (denaturation) and 60°C for 60s (annealing and extension).

| Table 1. Viral and bacterial pathogens detected using Taqman array cards (TAC) among non-fatal and fatal cases and asymptomatic controls, by age group , western Kenya, 2009-11 | | | | | | | | | | |
| --- | --- | --- | --- | --- | --- | --- | --- | --- | --- | --- |
|  | Total (non-fatal and fatal cases) | Non-fatal cases | | | Fatal cases | | | Asymptomatic controls | | |
|  | All ages | All ages | <5yrs | ≥5yrs | All ages | <5yrs | ≥5yrs | All ages | <5yrs | ≥5yrs |
|  | N=142 | N=72 | N=34 | N=38 | N=72 | N=34 | N=38 | N=72 | N=34 | N=38 |
| Viruses n (%) |  |  |  |  |  |  |  |  |  |  |
| Adenovirus | 13 (9) | 9 (13) | 4 (12) | 5 (13) | 4 (6) | 3 (9) | 1 (3) | 6 (8) | 3 (9) | 3 (8) |
| Influenza A | 11 (8) | 7 (10) | 4 (12) | 3 (8) | 4 (6) | 3 (9) | 1 (3) | 1 (1) | 1 (7) | 0 (0) |
| Influenza B | 1 (1) | 1 (1) | 1 (3) | 0 (0) | 0 (0) | 0 (0) | 0 (0) | 0 (0) | 0 (0) | 0 (0) |
| Human metapneumovirus | 1 (1) | 1 (1) | 0 (0) | 1 (3) | 0 (0) | 0 (0) | 0 (0) | 3 (4) | 2 (6) | 1 (3) |
| Parainfluenza virus type 1 | 1 (1) | 0 (0) | 0 (0) | 0 (0) | 1 (1) | 1 (3) | 0 (0) | 2 (3) | 0 (0) | 2 (5) |
| Parainfluenza virus type 2 | 0 (0) | 0 (0) | 0 (0) | 0 (0) | 0 (0) | 0 (0) | 0 (0) | 0 (0) | 0 (0) | 0 (0) |
| Parainfluenza virus type 3 | 8 (6) | 6 (8) | 4 (12) | 2 (5) | 2 (3) | 0 (0) | 2 (5) | 3 (4) | 2 (6) | 1 (3) |
| Respiratory syncytial virus | 12 (8) | 4 (6) | 2 (6) | 2 (5) | 8 (11) | 4 (12) | 4 (11) | 5 (7) | 0 (0) | 5 (13) |
| Enterovirus/Rhinovirus | 62 (44) | 29 (40) | 22 (65) | 7 (18) | 33 (46) | 20 (59) | 13 (34) | 28 (39) | 21 (62) | 7 (18) |
| *Total number of viruses* | *109* | *57* | *37* | *20* | *52* | *31* | *21* | *48* | *29* | *19* |
| Virus co-detection n (%) |  |  |  |  |  |  |  |  |  |  |
| Single virus | 59 (42) | 28 (39) | 17 (50) | 11 (30) | 31 (43) | 15 (44) | 16 (42) | 30 (42) | 16 (47) | 14 (37) |
| Two viruses | 15 (11) | 7 (10) | 6 (18) | 1 (3) | 8 (11) | 5 (15) | 3 (8) | 11 (15) | 8 (24) | 3 (8) |
| Three or more viruses | 9 (6) | 6 (8) | 4 (12) | 2 (5) | 3 (4) | 3 (9) | 0(0) | 0 (0) | 0 (0) | 0 (0) |
| *Total number of cases with at least one virus* | *83 (58)* | *41 (57)* | *27 (79)* | *14 (37)* | *42 (58)* | *23 (68)* | *19 (50)* | *41 (57)* | *24 (71)* | *17 (45)* |
| Bacteria n (%) |  |  |  |  |  |  |  |  |  |  |
| *Bordetella pertussis* 1 | 2 (1) | 0 (0) | 0 (0) | 0 (0) | 2 (3) | 1 (3) | 1 (3) | 1 (1) | 0 (0) | 1 (3) |
| *Hemophilus influenzae*-all types | 10 (7) | 5 (7) | 3 (9) | 2 (5) | 5 (7) | 4 (12) | 1 (3) | 41 (57) | 24 (71) | 17 (45) |
| *Mycoplasma pneumoniae* | 1 (1) | 1 (1) | 0 (0) | 1 (3) | 0 (0) | 0 (0) | 0 (0) | 0 (0) | 0 (0) | 0 (0) |
| *Streptococcus pneumoniae* | 85 (60) | 41 (57) | 28 (82) | 13 (34) | 44 (61) | 28 (82) | 16 (42) | 42 (58) | 28 (82) | 14 (37) |
| *Total number of bacteria* | *98* | *47* | *31* | *16* | *51* | *33* | *18* | *84* | *52* | *32* |
| Bacteria co-detection n (%) |  |  |  |  |  |  |  |  |  |  |
| Single bacterium | 75 (53) | 37 (51) | 25 (74) | 12 (41) | 38 (53) | 24 (71) | 14 (37) | 20 (28) | 6 (18) | 14 (37) |
| Two bacteria | 10 (7) | 5 (7) | 3 (9) | 2 (5) | 5 (7) | 3 (9) | 2 (5) | 32 (44) | 23 (68) | 9 (24) |
| Three or more bacteria | 1 (1) | 0 (0) | 0 (0) | 0 (0) | 1 (1) | 1 (3) | 0 (0) | 0 (0) | 0 (0) | 0 (0) |
| *Total number of cases with at least one bacteria* | *86 (61)* | *42 (58)* | *28 (82)* | *14 (37)* | *44 (61)* | *28 (82)* | *16 (42)* | *52 (72)* | *29 (85)* | *23 (61)* |
| Virus and bacteria co-detection n (%) |  |  |  |  |  |  |  |  |  |  |
| Virus alone | 23 (16) | 12 (17) | 6 (18) | 6 (16) | 11 (15) | 2 (6) | 9 (24) | 7 (10) | 1 (3) | 6 (16) |
| Bacteria alone | 26 (18) | 13 (18) | 7 (21) | 6 (16) | 13 (18) | 7 (21) | 6 (16) | 18 (25) | 6 (18) | 12 (32) |
| Mixed virus and bacteria | 60 (42) | 29 (40) | 21 (62) | 8 (21) | 31 (43) | 21 (62) | 10 (26) | 34 (47) | 23 (68) | 11 (29) |
| *Total number of cases with at least one virus and/or bacteria* | *109 (77)* | *54 (75)* | *34 (100)* | *20 (52)* | *55 (76)* | *30 (88)* | *25 (66)* | *59 (82)* | *30 (88)* | *29 (76)* |

| Table S2. Distribution of respiratory pathogens among cases (non-fatal and fatal) and corresponding asymptomatic controls in rural western Kenya, 2009-11. | | | | | | |
| --- | --- | --- | --- | --- | --- | --- |
|  | Non-fatal Cases (N=72) | Asymptomatic Controls (N=72) | OR(95% CI) | Fatal Cases (N=72) | Asymptomatic Controls (N=72) | OR(95% CI) |
| Respiratory pathogens |  |  |  |  |  |  |
| Viruses |  |  |  |  |  |  |
| Adenovirus | 9 (13) | 6 (8) | 2.00 (0.50-8.00) | 4 (6) | 6 (8) | 0.60 (0.14-2.51) |
| Influenza A | 7 (10) | 1 (1) | 3.00 (0.31-28.84) | 4 (6) | 1 (1) | 4.00 (0.45-35.79) |
| Influenza B | 1 (1) | 0 (0) | - | 0 (0) | 0 (0) | - |
| Human metapneumovirus | 1 (1) | 3 (4) | 0.33 (0.03-3.20) | 0 (0) | 3 (4) | - |
| Parainfluenza virus 1 | 0 (0) | 2 (3) | - | 1 (1) | 2 (3) | 0.5 (0.05-5.51) |
| Parainfluenza virus 2 | 0 (0) | 0 (0) | - | 0 (0) | 0 (0) | - |
| Parainfluenza virus 3 | 6 (8) | 3 (4) | 2.00 (0.50-8.00) | 2 (3) | 3 (4) | 0.67 (0.11-3.99) |
| Respiratory syncytial virus | 4 (6) | 5 (7) | 0.80 (0.21-2.98) | 8 (11) | 5 (7) | 1.75 (0.51-5.98) |
| Enterovirus/Rhinovirus | 29 (40) | 28 (39) | 1.08 (0.51-2.29) | 33 (46) | 28 (39) | 1.36 (0.68-2.71) |
| Bacteria |  |  |  |  |  |  |
| *Bordetella pertussis* types I and II | 0 (0) | 1 (1) | - | 2 (3) | 1 (1) | 2 (0.18-22.06) |
| ***Hemophilus influenzae*** | **5 (7)** | **41 (57)** | **0.08 (0.02-0.25)** | **5 (7)** | **41 (57)** | **0.05 (0.12-0.22)** |
| *Mycoplasma pneumoniae* | 1 (1) | 0 (0) | - | 0 (0) | 0 (0) | - |
| *Streptococcus pneumoniae* | 41 (57) | 42 (58) | 0.93 (0.45-1.93) | 44 (61) | 42 (58) | 1.15 (0.55-2.42) |
